# Estimation of input functions from dynamic [^18^F]FLT PET studies of the head and neck with correction for partial volume effects

**DOI:** 10.1186/2191-219X-3-84

**Published:** 2013-12-27

**Authors:** Sara L Hackett, Dan Liu, Anastasia Chalkidou, Paul Marsden, David Landau, John D Fenwick

**Affiliations:** 1 Gray Institute for Radiation Oncology and Biology, Department of Oncology, University of Oxford, Oxford OX3 7DQ, UK; 2 PET Imaging Centre, Guys and St Thomas’ Hospital, King’s College London, London SE1 7EH, UK; 3 Department of Oncology, Guys and St Thomas’ Hospital, King’s College London, London SE1 7EH, UK

**Keywords:** Input function, Partial volume effect, Simultaneous estimation

## Abstract

**Background:**

We present a method for extracting arterial input functions from dynamic [^18^F]FLT PET images of the head and neck, directly accounting for the partial volume effect. The method uses two blood samples, for which the optimum collection times are assessed.

**Methods:**

Six datasets comprising dynamic PET images, co-registered computed tomography (CT) scans and blood-sampled input functions were collected from four patients with head and neck tumours. In each PET image set, a region was identified that comprised the carotid artery (outlined on CT images) and surrounding tissue within the voxels containing the artery. The time course of activity in the region was modelled as the sum of the blood-sampled input function and a compartmental model of tracer uptake in the surrounding tissue.

The time course of arterial activity was described by a mathematical function with seven parameters. The parameters of the function and the compartmental model were simultaneously estimated, aiming to achieve the best match between the modelled and imaged time course of regional activity and the best match of the estimated blood activity to between 0 and 3 samples. The normalised root-mean-square (RMS_norm_) differences and errors in areas under the curves (AUCs) between the measured and estimated input functions were assessed.

**Results:**

A one-compartment model of tracer movement to and from the artery best described uptake in the tissue surrounding the artery, so the final model of the input function and tissue kinetics has nine parameters to be estimated. The estimated and blood-sampled input functions agreed well when two blood samples, obtained at times between 2 and 8 min and between 8 and 60 min, were used in the estimation process (RMS_norm_ values of 1.1 ± 0.5 and AUC errors for the peak and tail region of the curves of 15% ± 9% and 10% ± 8%, respectively). A third blood sample did not significantly improve the accuracy of the estimated input functions.

**Conclusions:**

Input functions for FLT-PET studies of the head and neck can be estimated well using a one-compartment model of tracer movement and TWO blood samples obtained after the peak in arterial activity.

## Background

The role of positron emission tomography (PET) in cancer staging and assessment of treatment response continues to grow [1]. The radiotracer 3^′^-deoxy- 3^′^-[^18^F]fluoro-thymidine (FLT) is a marker for cell proliferation as it is phosphorylated by thymidine kinase-1, which is selectively expressed in the S, G2 and M phases of the cell cycle [2-4]. Numbers of proliferating cells are more closely related to particular aspects of FLT uptake kinetics than to tracer uptake at a fixed time point, and quantitative information about tumour uptake kinetics can be extracted from dynamic FLT-PET images via compartment modelling, a process that requires the time course (‘time-activity curve’ (TAC)) of tracer concentration in arterial blood to be used as an input function.

The gold standard method used to determine blood TACs is continuous arterial sampling, which is invasive, requires expensive equipment and carries a small risk to patients [5]. Consequently, other sources of arterial input functions have been explored, such as TACs taken from the vicinity of large blood vessels in dynamic PET images [6,7]. However, accurate determination of input functions from PET images is hindered by the ‘partial volume effect’ (PVE), a term that refers to two distinct but related phenomena: spillover and the tissue-fraction effect [8]. Spillover is caused by the finite spatial resolution of the imaging system and manifests as a blurring of the imaged distribution of activity. The tissue-fraction effect arises because the activity reported in each PET voxel is an average of the corresponding volume, which usually comprises tissues of different types and activity concentrations.

The diameter of the largest blood vessels in the head and neck is around 5 mm [9], which is comparable to the resolution of PET scanners and the dimensions of PET voxels. Some methods used to extract input functions from PET images attempt to explicitly correct the PVE using anatomical information taken from computed tomography (CT) or magnetic resonance scans [10-12]. Other approaches, most commonly used in brain imaging, assume that the activity measured in a region represents a mixture of blood and surrounding tissue activities and implicitly correct the PVE by modelling the relative contributions of blood and tissue to the total imaged regional activity [13-16].

Models of both the input function and tracer movement between the blood vessel and the surrounding tissue can be simultaneously fitted to a total regional TAC taken from the vicinity of the blood vessel in the dynamic PET images. This technique has shown promise for the radiotracer 2-deoxy-2-[^18^F]fluoro-D-glucose (FDG) in human [13] and animal [15] studies, and for the radiotracers FLT [16] and ^18^F-fluoromisonidazole (Fmiso) [17] in human studies. In this approach, the input function is represented as a mathematical formula, and activity in the surrounding tissue is linked to it via a compartment model. The parameters of the input formula and compartment model are estimated simultaneously so that the combined modelled activity best matches the regional TAC obtained from the dynamic PET images. A challenge for simultaneous estimation is that the number of parameters may be large and a unique solution not identifiable. Further, the previous studies have used a variety of compartmental (and empirical) models to describe tracer movement between a vessel and the surrounding tissue. An optimal model, which best balances accurate description of the tissue TAC and parsimony of parameters, has not yet been established.

This work identifies an appropriate model of tracer movement, then uses the model to simultaneously estimate arterial input functions for bolus injections of FLT from TACs of carotid artery regions drawn on dynamic FLT-PET images. We also explore the use of small numbers of arterial concentration measurements obtained from blood samples to refine possible solutions and thus improve the precision of the resulting arterial TAC estimates. Optimal times for blood sampling have been assessed.

## Methods

Written informed consent was obtained from all participants, and the study was approved by the local Research Ethics Committee and UK Administrat0ion of Radioactive Substances Advisory Committee.

### Patient details and image acquisition

Six datasets were collected for four patients diagnosed with stage II-III head and neck squamous cell carcinoma. Patients were scanned at the PET imaging centre of St Thomas’ Hospital, London using a GE Healthcare Discovery VCT PET-CT scanner (Waukesha, WI, USA). After CT scanning of the head and neck region, patients were injected with 2.59 MBq/kg of [^18^F]FLT (up to a maximum of 185 MBq), and PET imaging begun. List-mode PET data were binned into 24 frames (8 × 15 s, 4 × 30 s, 6 × 60 s, 2 × 300 s and 4 × 600 s) for four of the dynamic image sets, while for two image sets, data were binned into 103 frames (30 × 2 s, 12 × 10 s, 6 × 20 s and 55 × 60 s).

Images were reconstructed using Fourier rebinning, and a two-dimensional filtered back projection algorithm to ensure quantitative accuracy [18]. Corrections for attenuation, scatter and dead time were performed during sinogram histogramming and reconstruction.

An image array of 128 × 128 × 47 voxels per frame was used, with dimensions of 5.47 × 5.47 × 3.27 mm^3^. The transaxial full-width half-maximum (FWHM) and full-width tenth-maximum (FWTM) values of the scanner at 1 cm along the transaxial axis were 4.9 and 9.5 mm, respectively, and the axial FWHM and FWTM were 4.8 and 10.6 mm, respectively. The voxel spacing of the co-registered CT images was 0.98 × 0.98 × 3.27 mm^3^.

Blood samples were collected through a cannula placed in the radial artery, and ^18^F activity was measured using an Allogg Arterial Blood Sampling System (Allogg AB, Mariefred, Sweden). For the first 24 min, samples were taken at a rate of 1/s. A further two samples were taken at 38 and 56 min post-injection. Blood-sampled input functions, *b*(*t*), were constructed from ^18^F concentrations in the samples.

### Modelling the imaged TAC of the arterial region using the blood-sampled input function

**Figure 1:**
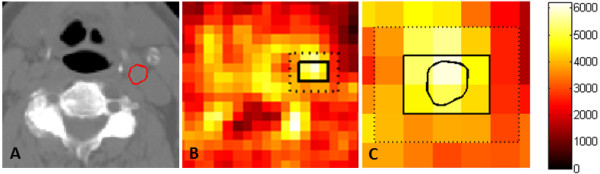
**Regions of interest in a PET image. (A)** A CT slice on which the carotid artery, outlined in red, was delineated. **(B)** The arterial voxels and external voxels on the corresponding PET image recorded at 2 min. The blood-sampled activity at this time was 12.3 kBq. The arterial voxels containing the carotid artery and surrounding tissue are delineated by the solid black line, and the carotid artery is outlined on the PET voxels in **(C)**. The external tissue, comprising the next layer of voxels out, is delineated by the dashed line.

For each dataset, the carotid artery was contoured on eight contiguous CT slices and the contours are transferred to the PET images. An example of the outlined carotid artery is shown in Figure 1. The corresponding PET voxels, also shown in Figure 1, that included the artery also comprised a significant fractional volume of extravascular tissue. The arterial voxels were identified on each of the eight slices, and the fractional volumes of artery and surrounding tissue, *v*_*a*_ and *v*_*tis*_, respectively, were determined. The carotid artery typically constituted 30% to 50% of the arterial voxel volume in the images used in this study. The total activity of the arterial voxels, *m*_tot_, can be modelled as

$$\begin{array}{lll} m_{\text{tot}}(t) & =v_{a}C_{a}(t)+v_{\text{tis}}C_{\text{tis}}(t)\; & \;\\ & =v_{a}C_{a}(t)+\left(1-v_{a}\right)\times f\left[C_{a}(t)\right]\; & \; \end{array}$$

where *C*_*a*_ and *C*_tis_ are models of the input function and the TAC of the surrounding tissue, respectively, and *f* is a compartmental (or empirical) model of the tissue TAC with input function *C*_*a*_.

However, the total regional activity recorded in the delineated PET voxels also comprises spill-in from the tissue in the next layer of voxels out, less the spill-out from the arterial voxels. Thus, the total activity also needs to be modified to correct for spillover, as described in the ‘Modelling of spillover’ section:

(1) $$m_{\text{tot}}(t)={\left[v_{a}C_{a}(t)+\left(1-v_{a}\right)\times f\left[C_{a}(t)\right]\right]}_{\text{spillover}}.$$

We have tested four different compartmental models: (a) irreversible uptake of tracer in a single non-vascular compartment, (b) reversible tissue uptake, (c) reversible uptake of tracer in the first non-vascular compartment from which tracer was irreversibly taken up in a second non-vascular compartment and (d) reversible uptake of tracer in the first and second non-vascular compartments. These models have one, two, four and five parameters, respectively. We have also tested a two-parameter, phenomenological model of tracer movement used by Backes et al. [16]. For each dataset, the five m_tot_ models were fitted to the mean activity, PET_tot_, recorded in the arterial voxels using the corresponding blood-sampled data as the input function for the compartmental model. The MATLAB function ‘lsqnonlin’ (The MathWorks, Natick, MA, USA) was used to fit m_tot_ to PET_tot_ by minimizing the objective function

(2) $$O=\overset{N}{\underset{i=1}{\sum }}w_{i}{\left(m_{\text{tot}}\left(t_{i}\right)-{\text{PET}}_{\text{tot}}\left(t_{i}\right)\right)}^{2}$$

where *t*_*i*_ is the time at the midpoint of the *i*th of *N* measurement intervals, and each weight *w*_*i*_ is inversely proportional to the variance of the imaged tracer concentration averaged over the *i*th measurement interval.

The optimal model was identified as that with the lowest Akaike information criterion corrected for small sample size (AIC_c_) [19]. The AIC_c_ values for all datasets are shown in Table 1. For all datasets, model B had the lowest AIC_c_ value, indicating that a compartmental model that accounted for tracer movement to and from the vessel was necessary and sufficiently complex to describe the data. The equation of state for model B is

(3) $$\frac{{\text{dC}}_{\text{tis}}}{\text{dt}}=K_{1}C_{a}(t)-k_{2}C_{\text{tis}}(t)$$

**Table 1:** **AIC**_**c **_**values of models**

**Dataset**			**Model AIC**_**c**_** value**		
	**A**	**B**	**C**	**D**	**Backes**
P1_1	19.1	5.7	9.7	11.7	12.9
P1_2	19.8	13.0	17.0	19.0	13.2
P2_1	33.1	31.9	35.8	37.8	40.5
P2_2	27.6	21.8	25.7	27.7	22.8
P3_1	7,386	1,966	1,969	1,971	4,929
P4_1	1,676	427	431	433	1,277

AIC_c_ values, calculated with simulation of spillover, are fitted to the *I*_tot_(*t*) data using the blood-sampled input function *b*(*t*).

where *K*_1_ and *k*_2_ are kinetics parameters describing the rate of tracer movement to and from the tissue, respectively.

### Simultaneous estimation of the input function and tissue TAC from PET_tot_

Having found the model *m*_tot_ which provides the best estimate of the activity recorded in the arterial voxels, we again fitted this model to *PET*_*tot*_ in order to extract the input function by minimizing the objective function given in Equation 2. In this process, it was assumed that the unknown input function is described by a mathematical model whose parameters are simultaneously estimated along with the kinetics parameters of the compartmental model. The input function *C*_*a*_ can conveniently and accurately be represented by the seven-parameter function [20]:

(4) $$\begin{array}{l} \;C_{a}(t)=\{\begin{array}{ll} 0 & \;\;\text{if}\;t<\;\tau \\ \left(A_{1}\left(t-\tau \right)-A_{2}-A_{3}\right)\exp\left(L_{1}\left(t-\tau \right)\right)\ldots \\ +A_{2}\exp\left(L_{2}\left(t-\tau \right)\right)+A_{3}\exp\left(L_{3}\left(t-\tau \right)\right) & \;\;\text{if}\;t\geq \;\tau \end{array} \end{array}$$

**Figure 2:**
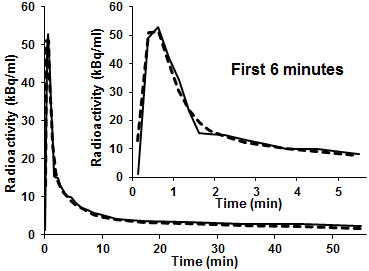
**Seven-parameter function fitted to blood-sampled input function.** A blood-sampled input function (solid line), measured for the corresponding PET dataset shown in Figure 1, and the fitted function (dashed line).

An example of a blood-sampled input curve is shown in Figure 2, together with a fit of Equation 4. Simpler mono- and bi-exponential models of the input function (both with a time-delay parameter for tracer delivery) were also tested but could not adequately describe the blood-sampled input functions. Thus, the final model of the total activity of the arterial pixels has nine parameters to be estimated: two kinetic parameters and seven input function parameters.

Blood samples obtained at a single time point were incorporated into the process of fitting *m*_tot_ to *PET*_*tot*_ by modifying the objective function to

(5) $$\;O=\overset{N}{\underset{i}{\sum }}w_{i}{\left(m_{\text{tot}}\left(t_{i}\right)\;-\;{\text{PET}}_{\text{tot}}\left(t_{i}\right)\right)}^{2}+\mathrm{\alpha .}\bar{w}{\left(b\left(t_{k}\right)\;-\;C_{a}\left(t_{k}\right)\right)}^{2}$$

where *b*(*t*_*k*_) is the measured arterial concentration at time *t*_*k*_ and $\bar{w}$ is the mean value of the weighting factors *w*_*i*_. The constant *α* is a ratio by which the squared difference between the estimated and measured blood concentrations has more influence on the fitting process than the average of the squared residual errors of the modelled *m*_tot_(*t*) values. A range of *α* values from 2 to 11 was considered and changes in the accuracy of the *C*_*a*_ models as a function of *α* were assessed using analysis of variance (ANOVA). The nine independent parameters of the full *m*_tot_ model are reduced to eight if the model is constrained to match a blood sample value at a particular time point. Thus, in order to avoid under- or over-emphasizing the fit at that time point, a reasonable *α* value would be around *N*×1/8 (that is, 3 or 13 for *N*=24 or 103), so that the weight given to the fit of the blood-sample point is one eighth that of the total weight of the fit of the model to *PET*_*tot*_(*t*). For additional blood samples, fitting was achieved by adding further terms to Equation 5, so that for three samples taken at times *t*_*k*_, *t*_*l*_ and *t*_*m*_, the objective function

$$\;\begin{array}{l} O=\sum_{i}^{N}\;w_{i}{\left(m_{\text{tot}}\left(t_{i}\right)-{\text{PET}}_{\text{tot}}\left(t_{i}\right)\right)}^{2}+\mathrm{\alpha .}\bar{w}{\left(b\left(t_{k}\right)\;-\;C_{a}\left(t_{k}\right)\right)}^{2}+\ldots \\ \;\mathrm{\alpha .}\bar{w}{\left(b\left(t_{l}\right)\;-\;C_{a}\left(t_{l}\right)\right)}^{2}+\mathrm{\alpha .}\bar{w}{\left(b\left(t_{m}\right)\;-\;C_{a}\left(t_{m}\right)\right)}^{2} \end{array}$$

was used. For two and three blood samples, reasonable *α* values would be 2*N*/(2×7) and 3*N*/(3×6) (3 and 4 respectively for *N*=24, and 14 and 17 respectively for *N*=103).

### Initial values of model parameters used for simultaneous estimation

The initial values of the kinetics parameters *K*_1_ and *k*_2_ used in the simultaneous estimation were the median values of the parameters obtained when Equation 1 (using model B) was directly fitted to *PET*_*tot*_ using *b*(*t*) as the input function. The initial values used for the input function parameters were the medians of those obtained by fitting Equation 4 to the *PET*_*tot*_(*t*) data and are termed *Init*_*PET*_. We also studied the use of the median values *Init*_*BSIF*_, obtained from directly fitting Equation 4 to the blood-sampled input function, as initial values for the estimated input function.

### Metrics used for evaluation of results

To evaluate agreement between a blood-sampled input function *b*(*t*) and a simultaneously estimated input function *C*_*a*_(*t*) we used a scale-independent measure of the root-mean-square (RMS) error

$${\text{RMS}}_{\text{norm}}={\left(\frac{1}{N}\frac{\sum_{i=1}^{N}{\left(C_{a}\left(t_{i}\right)-b\left(t_{i}\right)\right)}^{2}}{\sum_{i=1}^{N}{\left(b_{\text{direct}}\left(t_{i}\right)-b\left(t_{i}\right)\right)}^{2}}\right)}^{\frac{1}{2}}$$

where *b*_direct_(*t*) is the direct fit of Equation 4 to the blood-sampled input function *b*(*t*). The RMS _norm_ parameter therefore represents the goodness-of-fit of the estimated input function to the blood-sampled data relative to the goodness-of-fit of a direct fit of Equation 4 to the blood-sampled data.

Errors on areas under the curve (AUC) of the estimated input functions were also calculated. The input functions were divided into an early region, characterized by a sharp peak in activity, and a tail during which tracer concentrations in the blood and tissue gradually equilibrated. The boundary between the two regions was defined by the maximum value of the second derivative of *b*(*t*), typically around 6 min. The AUC error for each region was expressed as the modulus of the difference between the areas under the estimated and blood-sampled input functions, calculated as a percentage of the area under the blood-sampled input function.

### Modelling of spillover

Spillover was incorporated into the models of the total regional activity using the geometric transfer matrix (GTM) method [10]. This approach assumes that the activity is distributed over a number of regions, each with homogeneous concentration of activity. The GTM relates a vector of the true concentrations in the regions at time point *t*_*k*_, **C**_true_(*t*_*k*_), to a vector of measured concentrations **C**_meas_(*t*_*k*_), via

(6) $$GC_{\text{true}}\left(t_{k}\right)=C_{\text{meas}}\left(t_{k}\right)$$

where the elements *g*_*ij*_ of the matrix **G** represent the contribution of the true activity in region *i* to the activity recorded in region *j*. The GTM is usually inverted to recover the true concentrations in the regions of interest:

(7) $$C_{\text{true}}\left(t_{k}\right)=G^{-1}C_{\text{meas}}\left(t_{k}\right)$$

The activity recorded in the layer of voxels surrounding the arterial voxels, termed the external voxels, was significantly lower than the activity of the non-vascular tissue within the arterial voxels, termed the surrounding tissue, particularly around the peak in arterial activity concentration, as illustrated by Additional file 1: Figure S1.

Thus, three regions of interest were considered for the GTM method: the artery, the surrounding tissue and the external voxels. Anatomical information from the CT images was used to construct a high-resolution geometric model of each region of interest. The activity in each specific region was sequentially set to 1, and all other regions were set to 0. For each region *i*, this model was then convolved with a model of the point-spread function (PSF) of the PET scanner, and the intersection fraction of region *i* with each region *j* was calculated to generate the elements *g*_*ij*_.

The simultaneous estimation process aims to estimate the input function by fitting the model of Equation 1 to the total activity recorded in the delineated arterial voxels, which is affected by spillover. Equation 6 was therefore used to simulate the effects of spillover on the ‘true’ estimated concentrations of the artery and surrounding tissue concentrations before matching the total activity of these regions, *m*_tot_(*t*_*k*_), to that of the imaged region, *PET*_*tot*_(*t*_*k*_).

As the activity recorded in the arterial voxels also includes spill-out from the external voxels, the true concentration of activity in these voxels, **C**_ext_(*t*_*k*_), is required to calculate *m*_tot_(*t*_*k*_). **C**_ext_(*t*_*k*_) was estimated using Equation 7 to correct the activity measured in these voxels for spill-out from the arterial voxels. For this purpose, the activity in both the artery and surrounding tissue was set as the mean of the total activity in the arterial voxels. A vector of the estimated values of the arterial activity, surrounding tissue activity and the true activity in the external voxels was then multiplied by **G** to obtain a model of *m*_tot_(*t*_*k*_) with simulation of spillover. Models of *m*_tot_(*t*_*k*_) without simulation of spillover were also generated to examine the importance of explicitly simulating the effects of spillover on the accuracy of the estimated input functions.

The effects of the reconstruction algorithm and associated filters were modelled as a composite of two Gaussian functions fitted to the PSF of the PET scanner. This kernel was assumed to be stationary as all regions of interest lay within 5 cm of the centre of the transaxial field of view.

### Statistical analysis

ANOVA was used to study the influence of *α* on RMS _norm_ values of simultaneously estimated input functions. For simultaneous estimation using a single blood sample, input functions were generated for different sampling times *t*_*k*_, and median values and interquartile ranges of their RMS _norm_ values and AUC errors were tabulated, splitting the sampling times into ‘early’ (0 <*t*≤2 min), ‘intermediate’ (2 <*t*≤8 min) and ‘late’ (8 <*t*≤60 min) intervals. The interquartile range describes the change in a metric with sampling time *t*_*k*_ throughout an interval and also reflects the variability introduced by noise on the measured arterial tracer concentration.

The *F* test was used to compare residual errors for input functions generated using one versus two and two versus three blood samples. The appropriate (combination of) sampling times were identified as those times that resulted in the lowest median RMS _norm_ values for all six datasets. When two blood samples were used, median and interquartile ranges of RMS _norm_ and AUC errors were tabulated for the six possible combinations of early, intermediate and late blood-sampling intervals, and ANOVA was used to assess differences in these metrics between different combinations of sampling intervals. RMS _norm_ distributions of input functions generated with and without simulating spillover were also compared using ANOVA.

## Results and discussion

### Results

#### Simultaneous estimation of the arterial input function and surrounding tissue TAC

**Figure 3:**
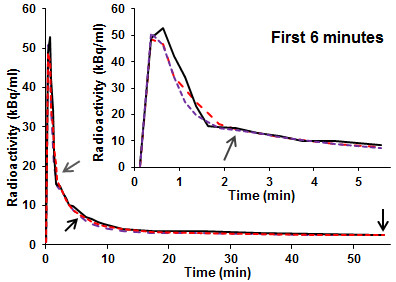
**Simultaneously estimated vs blood-sampled input functions: two and three blood samples.** A blood-sampled input function (solid black line) and input functions simultaneously estimated using two (dashed red line) or three (dashed purple line) blood samples taken at the times indicated by the arrows. The third blood sample was taken at the time indicated by the grey arrow.

Table 2 summarises the metrics of agreement between the blood-sampled and estimated input functions when blood samples were taken at (combinations of) times found to minimise RMS _norm_ values. Good agreement was achieved for all datasets (RMS _norm_ values of 1.0 to 1.5, median value 1.1) when arterial concentrations measured at two time points were used in the estimation process, but inclusion of a third sample did not significantly improve agreement for five of the six datasets (one *p* value of 0.002, all other *p* values >0.35). However, the input functions estimated without using any arterial concentration measurements showed notably worse agreement with the blood-sampled input functions (median RMS _norm_ 2.9) and the use of one measurement was not sufficient to achieve reasonable agreement (median RMS _norm_ 2.0). The *F* tests showed that inclusion of a second sample significantly improved the accuracy of five of the six estimated input functions datasets (*p* values <0.007). Figure 3 shows an example of input functions simultaneously estimated for one of the datasets using two and three concentration measurements and default *α* values of *N*×1/8, 2/ (2×7), 3/ (3×6) respectively (initial results showed no significant dependence of RMS _norm_ on *α*; all *p* values ≥ 0.35).

**Table 2:** Metrics for agreement of simultaneously estimated and blood-sampled input functions with simulation of spillover

	**Init**_**BSIF**_ **Number of samples**				**Init**_**PET**_ **Number of samples**			
	**0**	**1**	**2**	**3**	**0**	**1**	**2**	**3**
RMS _norm_	3.9	2.0 (0.7)	1.1 (0.5)	1.2	2.9	2.0 (0.3)	1.1 (0.4)	1.1
Peak AUC (%)	40	27 (8)	16 (7)	11	41	25 (9)	17 (9)	13
Tail AUC (%)	87	34 (15)	19 (11)	12	108	32 (9)	21 (15)	15

Median (median interquartile range) of RMS _norm_ values and errors of the area under the peak and tail of the estimated input function, as the moduli of the percentages of the areas under the blood-sampled input function (calculated with simulation of spillover). Samples were taken at late times (one sample only), combinations of intermediate and late times (two samples), and an early, intermediate and late time (three samples).

The accuracy of the input functions simultaneously estimated using zero or one arterial concentration measurements depends on the initial estimates of the model parameters; more accurate estimates were obtained by starting from *Init*_*BSIF*_ than *Init*_*PET*_. When only one arterial concentration measurement was used in the simultaneous estimation process, more accurate estimates of the input function were generally obtained using a measurement made in the late time interval (8 <*t*≤60 min). However, the use of one concentration measurement often improved the accuracy of the estimated input function only in the vicinity of the time point for which the measurement was obtained, whilst the accuracy over other intervals was unchanged or even worsened.

**Figure 4:**
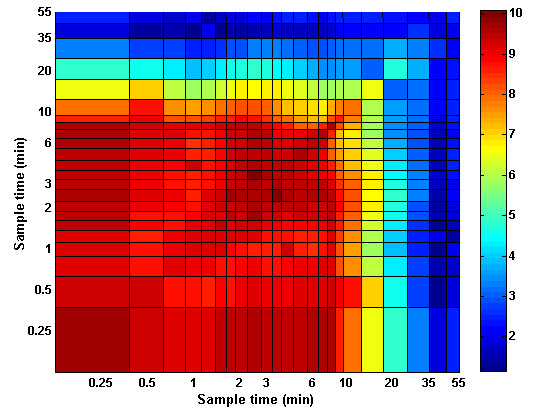
**Residual error of model as a function of sampling time.** RMS _norm_ value versus sampling times *t*_*k*_ and *t*_*l*_. The *Init*_*PET*_ values were used as initial values for the estimation process (with simulation of spillover).

In contrast, the accuracy of input functions estimated using two or three arterial concentration measurements was independent of initial parameter values. The lowest RMS _norm_ values were obtained using combinations of intermediate and late times of concentration measurement, as shown in Figure 4. Inclusion of the second concentration measurement taken in the intermediate interval improved the agreement between the blood-sampled and estimated input functions in the peak region without reducing the agreement in the tail region.

#### Spillover

For one, dataset, explicitly simulating spillover improved the agreement between input functions estimated with two blood samples (*p* value =0.03), but distributions of RMS _norm_ values were not significantly different for input functions estimated with and without spillover for the other five datasets. Metrics for agreement between blood-sampled and simultaneously estimated input functions obtained without simulation of spillover are summarized in Table 3. The (combinations of) sampling times are those used to generate the results presented in Table 2.

**Table 3:** Metrics for agreement of simultaneously estimated and blood-sampled input functions without simulation of spillover

	**Init**_**BSIF**_				**Init**_**PET**_			
	**Number of samples**				**Number of samples**			
	**0**	**1**	**2**	**3**	**0**	**1**	**2**	**3**
RMS _norm_	3.5	2.0 (0.4)	1.3 (0.9)	1.1	4.2	2.4 (0.4)	1.1 (0.4)	1.2
Peak AUC (%)	31	40 (11)	16 (11)	11	43	37 (6)	26 (9)	14
Tail AUC (%)	58	38 (9)	11 (24)	14	104	54 (15)	18 (14)	15

Median (median interquartile range) of RMS_norm_ values and errors of the area under the peak and tail of the estimated input function, as the moduli of the percentages of the areas under the blood-sampled input function (calculated without simulation of spillover).

## Discussion

It is clearly advantageous to generate input functions through non-invasive means, but the partial volume effect prevents direct extraction of accurate input functions from PET images. There is often a substantial tracer uptake in the tissue surrounding the arterial wall, and so any correction strategy for the PVE that ignores tracer uptake in the surrounding tissue is unlikely to accurately determine the input function. The method developed here enables accurate and robust estimation of input functions using image data and two blood samples. A one-compartment model that accounts for movement of the tracer to and from the artery is complex enough to describe the tracer kinetics but does not include unnecessary parameters that might hinder the estimation process. The complete model of the input function and tissue kinetics has nine parameters, seven for the input function model and two for the kinetics parameters, to be simultaneously estimated from the TAC of voxels that include the artery and two tracer concentrations obtained from blood samples taken at different time points.

When two arterial tracer concentration measurements, obtained at suitable times, are used in the simultaneous estimation process, the accuracy of the estimated input function is independent of the initial values chosen for the model parameters. If only one blood sample is used, the accuracy of the estimated input function depends on the choice of initial values, and the blood sample usually improves the accuracy of the estimated input function only around the time point at which the sample was taken. A combination of blood samples taken at intermediate and late times reduces the median RMS _norm_ values of the estimated input functions to only 10% greater than those obtained by directly fitting the input function model (Equation 4) to the arterial TACs obtained by continual blood sampling. Explicitly modelling the effects of spillover does not generally improve the accuracy of the input functions estimated using the proposed method.

The model described here works well for FLT PET head-and-neck images, from which TACs can be obtained for regions containing the carotid artery. However, it may not be appropriate for other tracers and/or imaged regions [12,21] for which the kinetics of tracer transport may differ. The process of model validation and selection, and exploration of the influence of direct measurements of blood concentrations on the accuracy of the estimated input functions, should be repeated for other radiotracers and imaging sites.

## Conclusions

Accurate descriptions of arterial input functions, delivered by bolus injection, can be obtained for FLT-PET through simultaneous estimation using a one-compartment model of tracer movement to and from the artery and two blood samples, taken at intermediate (2 <*t*≤8 min) and late (8 <*t*≤60 min) times.

## Abbreviations

AICc: Akaike information criterion (corrected for small sample size); ANOVA: Analysis of variance; AUC: Area under the curve; CT: Computed tomography; FDG: ^18^F-fluoro-deoxy-D-glucose; FLT: 3^*′*^-deoxy- 3^′^-[^18^F]fluoro-thymidine;; FWHM: Full-width half-maximum; FWTM: Full-width tenth-maximum; GTM: Geometric transfer matrix; PET: Positron emission tomography; PSF: Point-spread function; PVE: Partial volume effect; RMS: Root mean square; TAC: Time-activity curve.

## Competing interests

The authors declare that they have no competing interests.

## Authors’ contributions

JDF, DLa and PM conceived the study design. SLH and DLi performed the data analysis. PM and AC collected and processed imaging and blood sample data. SLH and JDF drafted the manuscript. All authors read and approved the final manuscript.

## Supplementary Material

Additional file 1 **Figure S1: Imaged activity of surrounding tissue and external voxels.** The activity in the surrounding tissue (solid black line) was determined at each time point by subtracting the blood-sampled input function, weighted by the fractional volume of the carotid artery in the arterial voxels, from the average imaged activity of these voxels (dashed black line). The average activity of the external voxels at each time point (solid blue line) was also measured from the images. The ‘true’ activity of the external voxels was estimated by substituting the averaged imaged activity of the arterial voxels as the activity of both the artery and surrounding tissue. Click here for file
