# Supplementary figures and images for: Estimation of input functions from dynamic [18F]FLT PET studies of the head and neck with correction for partial volume effects
Source: EJNMMI Res. 2013 Dec 27;3:84. doi: 10.1186/2191-219X-3-84 (PMC4109699; doi:10.1186/2191-219X-3-84)

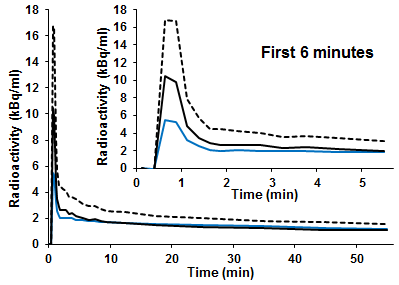

Supplement: Additional file 1 — Figure S1: Imaged activity of surrounding tissue and external voxels. The activity in the surrounding tissue (solid black line) was determined at each time point by subtracting the blood-sampled input function, weighted by the fractional volume of the carotid artery in the arterial voxels, from the average imaged activity of these voxels (dashed black line). The average activity of the external voxels at each time point (solid blue line) was also measured from the images. The ‘true’ activity of the external voxels was estimated by substituting the averaged imaged activity of the arterial voxels as the activity of both the artery and surrounding tissue. [file 2191-219X-3-84-S1.tiff]
